# Sequence variations of *ACVRL1* play a critical role in hepatic vascular malformations in hereditary hemorrhagic telangiectasia

**DOI:** 10.1186/s13023-020-01533-2

**Published:** 2020-09-22

**Authors:** Sophie Giraud, Claire Bardel, Sophie Dupuis-Girod, Marie-France Carette, Brigitte Gilbert-Dussardier, Sophie Riviere, Jean-Christophe Saurin, Mélanie Eyries, Sylvie Patri, Evelyne Decullier, Alain Calender, Gaëtan Lesca

**Affiliations:** 1 Hospices Civils de Lyon, Service de Génétique, Groupement Hospitalier Est, 69677 Bron, France; 2 Service de Biostatistique-Bioinformatique, plateforme de séquençage à haut débit, Hospices Civils de Lyon, Lyon, France; 3 Université Claude Bernard Lyon 1, Université de Lyon, Villeurbanne, France; 4 CNRS UMR 5558, Laboratoire de Biométrie et Biologie Evolutive, Equipe Biotatistique-Santé, F-69100 Villeurbanne, France; 5 Centre de Référence National pour la maladie de Rendu-Osler, Groupement Hospitalier Est, Bron, France; 6 Service de Radiologie, Hôpital Tenon, Paris, France; 7 Service Génétique, CHU de Poitiers, Poitiers, France; 8 EA3808, Université de Poitiers, Poitiers, France; 9 CHU de Montpellier, Service de Médecine Interne, Hôpital St Eloi, Montpellier, France; 10 Hospices Civils de Lyon, Service de Gastro-Entérologie, Hôpital E. Herriot, Lyon, France; 11 Assistance Publique-Hôpitaux de Paris, Département de Génétique, GH Pitié-Salpêtrière, Paris, France; 12 CHU la Milétrie, Laboratoire de Génétique, Poitiers, France; 13 Unité de recherche clinique du pole IMER of the Hospices Civils de Lyon, Lyon, France; 14 Equipe EA7426, Immunopathologie des voies respiratoires, Université Lyon 1, Lyon, France

**Keywords:** HHT, Rendu-Osler, *ACVRL1*, Modifier gene, Hepatic arteriovenous malformation

## Abstract

**Background:**

Hereditary Hemorrhagic Telangiectasia (HHT) is an autosomal dominant disorder characterized by multiple telangiectases and caused by germline disease-causing variants in the *ENG (HHT1)*, *ACVRL1 (HHT2)* and, to a lesser extent *MADH4* and *GDF2*, which encode proteins involved in the TGF-β/BMP9 signaling pathway. Common visceral complications of HHT are caused by pulmonary, cerebral, or hepatic arteriovenous malformations (HAVMs). There is large intrafamilial variability in the severity of visceral involvement, suggesting a role for modifier genes. The objective of the present study was to investigate the potential role of *ENG*, *ACVRL1*, and of other candidate genes belonging to the same biological pathway in the development of HAVMs.

**Methods:**

We selected 354 patients from the French HHT patient database who had one disease causing variant in either *ENG* or *ACVRL1* and who underwent hepatic exploration. We first compared the distribution of the different types of variants with the occurrence of HAVMs. Then, we genotyped 51 Tag-SNPs from the Hap Map database located in 8 genes that encode proteins belonging to the TGF-β/BMP9 pathway (*ACVRL1, ENG, GDF2, MADH4, SMAD1, SMAD5, TGFB1, TGFBR1*), as well as in two additional candidate genes (*PTPN14* and *ADAM17*). We addressed the question of a possible genetic association with the occurrence of HAVMs.

**Results:**

The proportion of patients with germline *ACVRL1* variants and the proportion of women were significantly higher in HHT patients with HAVMs. In the HHT2 group, HAVMs were more frequent in patients with truncating variants. Six SNPs (3 in *ACVRL1*, 1 in *ENG*, 1 in *SMAD5*, and 1 in *ADAM17*) were significantly associated with HAVMs. After correction for multiple testing, only one remained significantly associated (rs2277383).

**Conclusions:**

In this large association study, we confirmed the strong relationship between *ACVRL1* and the development of HAVMs. Common polymorphisms of *ACVRL1* may also play a role in the development of HAVMs, as a modifying factor, independently of the disease-causing variants.

## Background

Hereditary Hemorrhagic Telangiectasia (HHT, Rendu-Osler-Weber syndrome, ORPHA 774) is an autosomal dominant systemic disease whose primary pathogenic expression is arteriovenous malformation. Common manifestations are recurrent epistaxis beginning in childhood and cutaneous telangiectases, which are clusters of abnormally dilated capillaries. Visceral complications include intestinal bleeding and pulmonary (PAVM), hepatic (HAVM) and central nervous system arteriovenous malformations [1].

HHT is related to deleterious variants in 2 major genes: *ENG* (OMIM 131195) that encodes endoglin and which is located on chromosome 9q and causes HHT1 (OMIM 187300), and *ACVRL1* (OMIM 601284) that encodes activin receptor-like kinase type 1 (ALK1), which is located on chromosome 12q and causes HHT2 (OMIM 600376) [2, 3]. Disease-causing variants in the *MADH4* gene (OMIM 60093), which encodes SMAD4 and is located on chromosome 18q, have been found in patients with juvenile polyposis and HHT symptoms [4]. Furthermore, missense variants in *GDF2* gene (OMIM 605120), which encodes bone morphogenetic protein-9 (BMP9) and is located on chromosome 10q, have been found in 3 individuals with a vascular phenotype overlapping with HHT, characterized by epistaxis and telangiectasia without solid-organ involvement [5]. About 5–10% of the patients fulfilling the HHT clinical features do not have a germline pathogenic variant in one of these genes [6].

Mutations in two other genes, *RASA1* (OMIM 139150) and *EPHB4* (OMIM 600011), were reported in patients with overlapping, but clinically distinct disorders, named capillary malformation-arteriovenous malformation 1 (OMIM 608354) and 2 (OMIM 618196), respectively [7, 8].

Endoglin and ALK1 are predominantly expressed in endothelial cells and are receptors of the transforming growth factor-β (TGF-β) family that mediates responses to BMP9, and to a lesser extent, to BMP10 and TGF-β1/β3 [9, 10]. Both endoglin and ALK1 bind BMP9 and, in association with BMPR2, trigger signaling pathways mainly via SMAD1 and SMAD5, which form heteromeric complexes with SMAD4 and translocate into the nucleus where they regulate the transcriptional activity of target genes.

HAVMs consist of arteriovenous shunting responsible for high-output cardiac failure, ascites, and biliary ischemia with sepsis. Liver transplantation remains the main treatment option in advanced stages although new treatments such as anti-vascular endothelial growth factor therapies are emerging [11, 12]. The higher prevalence of HAVMs in patients with germline pathogenic *ACVRL1* variants, compared to *ENG*, has been suggested by several studies [13–16] and confirmed by a prospective study including 102 HHT patients [17]. In the latter study, based on data from hepatic Doppler sonography, 71% patients had HHT2, and 63% of these had HAVMs.

The wide intrafamilial clinical variability in HHT, including liver involvement, suggests a role for modifier genes, in addition to the *ENG* or *ACVRL1* disease-causing variants. The objective of the present study was to investigate the potential role of genetic variations in *ACVRL1*, *ENG*, and a selection of other genes encoding proteins of the TGFβ/BMP9 pathway in the development of HAVMs.

## Methods

### Patients

This is an non-interventional study including DNA from 354 patients with a diagnosis of HHT confirmed by molecular genetics (107 with *ENG* disease-causing variants and 247 with *ACVRL1* disease-causing variants) and who benefited from systematic hepatic evaluation by ultrasound Doppler (*n* = 247), contrast abnominal CT (*n* = 51), or both exams (*n* = 56). The patients were collected through the three laboratories involved in HHT genetic diagnosis in France: Lyon (*n* = 248), Paris (*n* = 91), and Poitiers (*n* = 15). The number of patients with *MADH4* disease-causing variants was too low (< 30) for statistical analyses. Patients had given written consent for the study of genetic factors involved in HHT according to the French bioethics law. Genetic and clinical data have been collected in the French HHT patient database (CIROCO) from 2005 to 2013.

Patients were classified into three groups according to clinical data or to liver imaging (if asymptomatic) that has previous shown to reflect disease severity [17]: i) group 1 had no liver abnormality: diameter of the common hepatic artery < 6.5 mm (which was the most commonly indicated measure in hepatic exploration), maximum velocity in the hepatic artery < 120 cm/s, ii) group 2 had isolated enlargement of the diameter (between 6.5 and 10.5) or of the velocity of the hepatic artery (≥ 120 cm/s), iii) group 3 had either a common hepatic artery diameter higher than 10.5 cm, heart failure due to hepatic shunt, or had undergone liver transplantation for severe liver involvement.

### Analysis of disease-causing *ENG* and *ACVRL1* variants

DNA was extracted from patient blood according to standard procedures. *ENG* and *ACVRL1* were analyzed by sequencing on a 3130XL sequencer (Thermo Fisher Scientific, Illkirch-Graffenstaden, France). Large rearrangements were identified by Quantitative Multiplex PCR Short Fragments (QMPSF) or Multiplex Ligation PCR Amplification (MLPA) using the P093 Salsa MLPA HHT/PPH1 probe set (MRC-Holland, Amsterdam, The Netherlands) on a 3130XL sequencer. The detailed description of analysis has been previously reported [18].

### SNP selection and genotyping

As potential candidate modifier genes for HHT, we selected *ACVRL1*, *ENG* and *MADH4*, the ligands (*BMP9/GDF2* and *TGFB1*) and the receptor of the TGF-β signaling pathway (*TGFBR1*)*,* and two main transcription factors implicated in the TGF-β signaling pathway (*SMAD1* and *SMAD5*)*.* Forty-eight Tag-SNPs from the HapMap project were chosen using Haploview V4.1 software (https://www.broadinstitute.org/haploview/haploview) [19]. All SNPs with a maximum r2 value of 0.8 (pairwise tagging approach) and a minor allele frequency (MAF) of at least 5% in CEU HapMap individuals (https://ftp.ncbi.nlm.nih.gov/hapmap/genotypes/2010-05_phaseIII/hapmap_format/polymorphic/) were selected: *ACVRL1* (rs7956340, rs11169953, rs11169954, rs1150051, rs2277383, rs706819, rs2293092), *ENG (*rs10987759, rs4836585, rs16930129, rs10121110, rs2005129, rs11792480, rs17557600, rs3739817, rs10819309, rs10987746, rs1330684), *GDF2* (rs3758496, rs12252199, rs9971293, rs9421799), *MADH4* (rs10502913, rs948588, rs12458752), *SMAD1* (rs6537355, rs959641, rs2118438, rs714195, rs1016792, rs13120142, rs12505085), *SMAD5* (rs5008734, rs746994, rs746993, rs17749249), *TGFB1* (rs2241715, rs4803455, rs8110090, rs11466338, rs11466345), and *TGFBR1* (rs10988705, rs10819638, rs6478974, rs10739778, rs10512263, rs928180, rs10733710).

In addition, we also genotyped two SNPs of the *PTPN14* gene (rs2936018 and the Tag-SNP rs3002297 chosen with Haploview) and one SNP in the *ADAM17* gene that have been shown to be associated with PAVMs in HHT1 patients [20, 21].

For genotyping, DNA was amplified by PCR (PCR conditions available upon request). Analysis of 36 SNPs was performed by High Resolution Melting (HRM) on a Light Scanner Instrument according to the manufacturer’s protocol (Idaho Technology, Salt Lake City, UT, USA) when heterozygous genotypes could be easily distinguished from the homozygous one. For the 15 other SNPS, since HRM was not conclusive, analysis was performed by direct sequencing on a 3130XL sequencer.

### Statistical analysis

#### Identification of modifier genes

In order to look for modifier gene for hepatic involvement in HHT, patients of group 1 (no liver disease) were compared to patients of group 2 + 3 (mild or severe hepatic involvement). The association between liver disease severity and the different SNPs was tested using a mixed-effects logistic regression model including sex, gene responsible for HHT (*ENG* or *ACVRL1*) and age at diagnosis of hepatic abnormality for patients of groups 2 and 3, or age at the last normal hepatic exploration for patients of group 1 (referred to as “age at hepatic screening” in the following sections) as covariates.

To improve the convergence of the model, age at hepatic exploration was centered. An individual random effect was added to take family relationships into account. The analysis was performed using the R package pedigreemm [22].

In a first step, all 51 SNPs were tested in turn. Their genotypes were modeled using an additive effect (genotypes were coded 0 for homozygous for the major allele, 1 for heterozygous and 2 for homozygous for the minor allele). As many tests were performed, the risk of false positive results was controlled using the False Discovery Rate (FDR) [23]. For all SNPs with *p* ≤ 0.05 (prior to the correction for multiple testing), a second model was fitted to estimate separately the effect of being homozygous for the minor allele or heterozygous compared to the reference (homozygous for the major allele).

#### Analysis of clinical variables

The effect of clinical variables (sex, age at hepatic screening, type of pathogenic variant, gene responsible for HHT) on the phenotype (groups 1, 2, and 3) was tested with two sided Cochran-Armitage trend tests (R package DescTools) for qualitative data or one-way analysis of variance for quantitative data. The proportion of missense variant in HHT1 and HHT2 was compared with Pearson Chi-Squared test. Some patients being related, the independence assumption may not exactly be met so the effect of these variables was confirmed in multivariate mixed-effect logistic regression models. In addition, when the type of variant is studied, there might be a bias due a founder mutation [24] that was found in 55 HHT2 patients. Analyses were thus performed both with and without these 55 patients.

## Results

### Clinical data

A total of 354 patients were analyzed: 206 females and 148 males. The proportion of females and the age of patients at hepatic exploration increased significantly in function of the severity of HAVM (Table 1). This was also found in mixed logistic models (*p* < 10^− 5^ for age at hepatic screening, and *p* < 10^− 3^ for sex).

**Table 1:** Clinical characteristics in the three phenotype groups of hepatic involvement

	Group 1	Group 2	Group 3	Total patients	*p*-value
Number of patients	123	138	93	354	
Number of females (% in the group)	55 (55.3%)	84 (60.9%)	67 (72.0%)	206 (58.2%)	4.35 × 10^−5^
Mean age at hepatic screening ± standard deviation	43.4 ± 15.5	51.5 ± 14.5	57.6 ± 13.0	50.3 ± 15.5	2.5 × 10^−11^
HHT2 (% of the cohort)	60 (48.8%)	104 (75.4%)	83 (89.2%)	247 (69.8%)	5.85 × 10^−11^

### Distribution of the *ACVRL1* and *ENG* disease-causing variants

On a total of 107 HHT1 patients, 44 (41.1%) had hepatic involvement, including 10 individuals of group 3. There were 247 HHT2 patients: 187 (75.7%) had hepatic disease including 83 of group 3. The proportion of HHT2 patients increased significantly according to the severity of HAVM (Table 1 and *p* < 10^− 5^ in the mixed logistic model).

The distribution of the types of disease-causing variants for *ACVRL1* (HHT2) and *ENG* (HHT1) was different: the proportion of missense was higher in the *ACVRL1* group (Table 2, all patients: *p* < 0.0002, founder mutation bias removed: *p* < 1,7 × 10^− 8^) in accordance with data from the international mutation database (http://arup.utah.edu/database/HHT).

**Table 2:** Type of variant according to the disease-causing gene

	Type of variant						
	Missense	In-frame indels	Splice-site	Non sense	Frameshift	Large deletion or duplication	Total
ENG* (HHT1)	25 (24.0%)	4 (3.8%)	16 (15.3%)	20 (19.2%)	33 (30.8%)	6 (5.6%)	104
*ACVRL1* (HHT2)	112 (45.3%)	4 (1.6%)	19 (7.7%)	25 (10.1%)	85 (34.4%)	2 (0.8%)	247

* 3 *ENG* variants which affected the initiation codon were excluded because their effect was not demonstrated

**Fig. 1:**
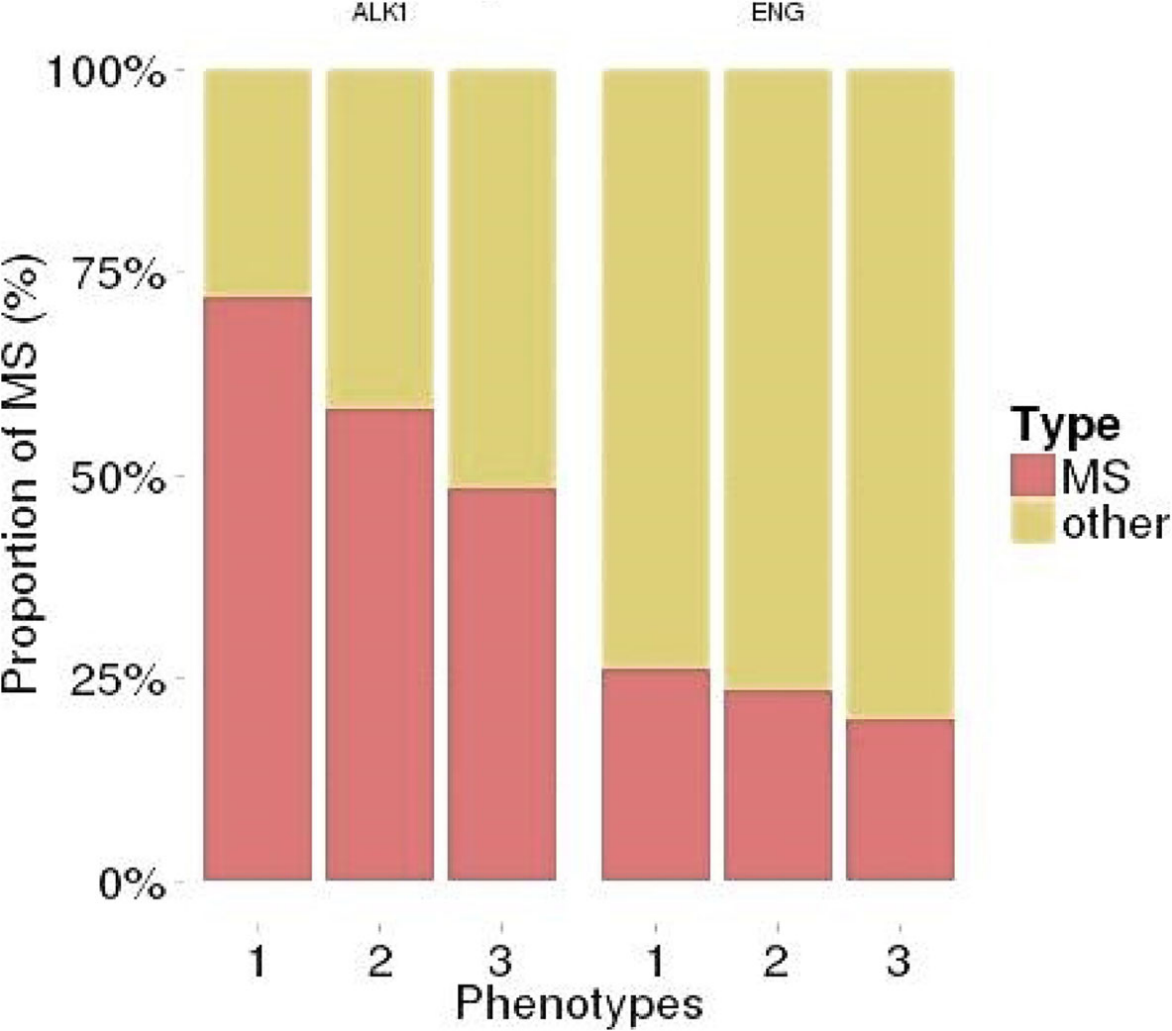
Proportion of missense variants (MS) and other variants (frameshift, stop and splice-site variants) according to the severity of hepatic involvement

In the HHT2 group, the proportion of truncating variants increased with the severity of liver involvement. The increase was not statistically significant when all HHT2 patients were considered (*p* = 0.052) but when the bias due to the founder mutation is removed, it is significant (*p* = 0.01254) phenotype (Fig. 1).

### Association with modifier genes

A significant association (*p* = 0.0001) was found between an *ACVRL1* SNP (rs2277383) and HAVM. This remained significant after correction for multiple testing with the FDR (*p* = 0.0052). There was also an association between HAVM and 3 other *ACVRL1* SNPs (rs11169953, rs1150051, rs2293092), one *ENG* SNP (rs3739817), one *SMAD5* SNP (rs5008734), and one *ADAM17* SNP (rs10495565), but none of them achieved significance after correction for multiple testing: an additional table file shows this in more details [Additional file 1].

When we analyzed the data within each HHT genetic subgroup, rs2277383 was not significantly associated with HAVM in the HHT1 patients. On the other hand, one *ACVRL1* SNP (rs1150051) and one *ADAM17* SNP (rs10495565) were associated with HAVM in HHT1 patients, but the association did not reach significance after correction for multiple testing by FDR. In contrast, in HHT2 patients, there was a significant association between rs2277383 and HAVM (*p* = 10^− 4^ before correction for multiple testing and *p* = 0.026 after correction) and an association with 2 other *ACVRL1* SPNs (rs11169953, rs2293092), and the same *SMAD5* SNP (rs5008734), which did not reach significance after correction for multiple testing.

## Discussion

In the present study we investigated the role of different genetic factors in the occurrence of HAVM in HHT patients. We confirmed the more severe hepatic involvement in females than in males, already observed in previous studies, with a growing proportion of women with the severity of hepatic disease (Table 1) [15, 16, 25]. One striking characteristic of HHT is the wide interfamilial and intrafamilial variability in the clinical expression of visceral AVMs. Interfamilial variability suggests that some disease-causing variants may have different consequences compared to others while intrafamilial variability is rather in favor of a role for additional factors, such as modifier genes.

We first studied the relationship between the germline pathogenic variant and the risk of HAVM. In a previous study of genotype-phenotype correlation in HHT we observed a higher frequency of hepatic involvement in HHT2 patients compared to HHT1 patients; although the difference did not reach statistical significance [15], it was supported by other studies [16, 26]. In the previous study only 57% (197/343) of the patients had systematic hepatic exploration and the criteria for hepatic involvement and severity were not well defined at that time [15], whereas, in this study, hepatic exploration following the criteria defined in 2008 was performed in all patients [17]. Clinical records were revisited and patients were classified into 3 severity groups. Based on this improved classification of patients we could confirm the higher frequency of HAVMs in HHT2 patients. Among HHT2 patients, the proportion of truncating variants increased with the severity of liver involvement. Significance was reached when we excluded the patients with the c.1112dup/p.Thr372fs variant, which results from a regional founder effect and may introduce confounding factors. No association with the type of variant was observed for the HHT1 group.

The large intrafamilial variability of HAVM in HHT suggests a role for modifier genes or for non-genetic factors. The higher prevalence of hepatic involvement in females, which is well known and observed herein, suggests a role for hormonal factors or other sex-determined genetic factors. However, it is of note that previous studies, based on immunochemistry analyses of oestrogen and progesterone receptors expression in patients with HHT has brought controversial results and do not support a role for hormonal receptors, at least in the mucosa from nasal telangiectases [27, 28].

Modifying genetic factors may be located in trans, i.e. on the allele of *ENG* or *ACVRL1,* which does not carry the disease-causing pathogenic variant or in a different gene. We tested the hypothesis of a modifier role for *ACVRL1*, *ENG*, and six other genes encoding proteins of the TGFβ/BMP9 pathway. Six SNPs (3 in *ACVRL1*, 1 in *ENG*, 1 in *SMAD5*, and 1 in *ADAM17*) were significantly associated with HAVMs but after correction for multiple testing, only one remained significantly associated (rs2277383). This SNP is located in the last intron of *ACVRL1* and is not predicted to affect splicing. The frequency of HAVMs was lower in HHT2 patients with the less frequent allele (G). Since this SNP is located in intron 9, 155 bases before exon 10 (c.1378-155 T/G) this association could be due to linkage disequilibrium with the c.1112dup founder pathogenic variant that was present in 53 patients (21%). In order to test this hypothesis, we performed another analysis excluding these patients. The association between this SNP and HAVM was still highly significant. These results suggest that the (G) allele could be a protective factor towards hepatic involvement, at least in HHT2 patients. This SNP could have an effect on the regulation of expression of the mutated allele of *ACVRL1.* Alternatively, it could be in linkage-disequilibrium with another SNP with a functional effect. A recent study suggested an association between the *ACVRL1* SNP (c.314-35A > G) and the occurrence of pulmonary and hepatic involvement in HHT1 patients [29]. This is rather in favor of the hypothesis of common mechanisms for the development of arteriovenous malformation in different organs. In that study, however, hepatic involvement was determined according to clinical screening only and no systematic echography evaluation was performed as was the case herein. This methodological difference may have influenced the results since liver involvement is age-dependent in HHT and evolved asymptomatically for many years.

In the present study we also included two SNPs of *PTPN14* and 1 SNP of *ADAM17*, which were previously suggested to play a role in HHT [20, 21]. *PTPN14* is located in a region orthologous to mouse *Tgfbm2* that is a modifier locus for the phenotype of *Tgfb*^*−/−*^ mice [30]. Association of two SNPs of this gene with the occurrence of PAVMs was reported in a cohort of 721 Dutch patients and replicated in a cohort of 222 French patients [20]. In the present study we did not detect any association between these two SNPs and hepatic involvement. *ADAM17* is located in a region orthologous to *Tgfbm3b*, another modifier locus for the *Tgfb*^*−/−*^ mice. Three SNPs of this gene were shown to be associated with the presence of PAVMs in HHT1 patients in the Dutch and French cohorts [21]. In the present study, we found a trend towards an association between HAVM and the common (G) allele of the *ADAM17* SNP (rs10495565) in HHT1 but not in HHT2 patients. Functional data provided supporting evidence for *PTPN14* involvement in the endoglin/ALK1 biological pathways in the lung [31]. The latter study also found that genetic variations in the wild-type *ENG* allele, inherited from the unaffected parent, may modify the risk for pulmonary AVMs in HHT1 patients. The pulmonary AVM-at-risk allele *ENG*-rs10987746-C associates with a slight but significant reduction in *ENG* transcript levels in human lymphoblastoid cell lines, providing supporting evidence that genetic variation within this gene may influence its own expression. In the present study, we also found a trend towards an association of one *ENG* SNP (rs3739817) with HAVM in the whole HHT group, but not in the HHT1 or HHT2 subgroups. A recent study in 752 HHT patients failed to find significant association for candidate genes with arteriovenous malformations of different organs, including HAVMs [32]. However, it is of note that the characterization of liver involvement was different in that study, based on hepatic or cardiac symptoms and signs only. The data presented herein suggest that the possible effects of modifier genes could be different according to the genetic type of HHT (HHT1 versus HHT2), resulting from a yet unknown mechanism. The identification of genetic factors that influence the clinical outcome of a disease with wide intrafamilial and interfamilial clinical variability, such as HHT, may lead to a better understanding of pathophysiological mechanisms and to pave the way for the development of targeted treatments.

## Limitations of the study

The fact that the significant association between the ANP rs2277383 and HAMV was not observed in the HHT1 subgroup may probably be due to the lower number of patients. The present results need to be confirmed by studies performed in larger cohorts of patients or supported by biological experiments on cellular or animal models.

## Conclusion

Our data confirm that *ACVRL1* variants are highly associated with the risk of HAVMs, and that this risk may be higher in HHT2 patients. In addition to the disease-causing variant, common polymorphisms of *ACVRL1* may also play a role in the modulation of this risk, at least in the HHT2 group. These results emphasize the major role of *ACVRL1* in the development of HAVMs.

## Supplementary information

**Additional file 1.**

## Data Availability

The datasets used and/or analyzed during the current study are available from the corresponding author on reasonable request.
